# MRPS31 loss is a key driver of mitochondrial deregulation and hepatocellular carcinoma aggressiveness

**DOI:** 10.1038/s41419-021-04370-8

**Published:** 2021-11-12

**Authors:** Seongki Min, Young-Kyoung Lee, Jiwon Hong, Tae Jun Park, Hyun Goo Woo, So Mee Kwon, Gyesoon Yoon

**Affiliations:** 1 Department of Biochemistry, Ajou University School of Medicine, Suwon, 16499 Korea; 2 Department of Biomedical Sciences, Graduate School, Ajou University, Suwon, 16499 Korea; 3 Department of Physiology, Ajou University School of Medicine, Suwon, 16499 Korea

**Keywords:** Cancer genomics, Metastasis, Tumour biomarkers

## Abstract

**Abstract:**

Deregulated mitochondrial energetics is a metabolic hallmark of cancer cells. However, the causative mechanism of the bioenergetic deregulation is not clear. In this study, we show that somatic copy number alteration (SCNA) of mitoribosomal protein (MRP) genes is a key mechanism of bioenergetic deregulation in hepatocellular carcinoma (HCC). Association analysis between the genomic and transcriptomic profiles of 82 MRPs using The Cancer Genome Atlas-Liver HCC database identified eight key SCNA-dependent MRPs: *MRPS31*, *MRPL10*, *MRPL21*, *MRPL15*, *MRPL13*, *MRPL55*, and *DAP3*. *MRPS31* was the only downregulated MRP harboring a DNA copy number (DCN) loss. *MRPS31* loss was associated specifically with the DCN losses of many genes on chromosome 13q. Survival analysis revealed a unique dependency of HCC on the *MRPS31* deficiency, showing poor clinical outcome. Subclass prediction analysis using several public classifiers indicated that *MRPS31* loss is linked to aggressive HCC phenotypes. By employing hepatoma cell lines with SCNA-dependent MRPS31 expression (JHH5, HepG2, Hep3B, and SNU449), we demonstrated that MRPS31 deficiency is the key mechanism, disturbing the whole mitoribosome assembly. MRPS31 suppression enhanced hepatoma cell invasiveness by augmenting MMP7 and COL1A1 expression. Unlike the action of MMP7 on extracellular matrix destruction, COL1A1 modulated invasiveness via the ZEB1-mediated epithelial-to-mesenchymal transition. Finally, MRPS31 expression further stratified the high COL1A1/DDR1-expressing HCC groups into high and low overall survival, indicating that MRPS31 loss is a promising prognostic marker.

**Significance:**

Our results provide new mechanistic insight for mitochondrial deregulation in HCC and present MRPS31 as a novel biomarker of HCC malignancy.

## Introduction

Mitochondria are essential cytoplasmic organelles that generate and supply most of the cellular energy required for maintaining the cell’s biochemical activities. Mitochondrial energy generation is accomplished by the oxidative phosphorylation (OXPHOS) system, which is composed of five enzymatic complexes: complex I, II, III, IV, and V. Most of the OXPHOS protein components originate from and are expressed by nuclear DNA (ncDNA), but 13 core proteins are encoded by the mitochondrial DNA (mtDNA) and, therefore, are exclusively expressed by the mitochondrial transcription and translation machinery in the mitochondria matrix [1]. Mitochondrial ribosomes (mitoribosomes) are the specialized macrostructures that govern the translation of the 13 mtDNA-encoded proteins. The protein portion of the mitoribosome is formed by 82 ncDNA-encoded mitoribosomal proteins (MRPs), whereas two structural ribosomal RNAs (rRNAs) come from mtDNA [2]. Therefore, well-balanced expression and assembly of the 82 MRPs and 2 rRNAs must be critical to the expression of the 13 proteins and the resultant OXPHOS bioenergetic activity.

Dysregulation of mitochondrial bioenergetics has been recognized as a key metabolic hallmark of malignancy [3]. However, the mechanisms underlying how mitochondrial dysregulation is acquired during tumor development and regulates the tumor cell activity remain unclear. Recently, PTCD3, also named MRPS39, was demonstrated to be critical in the maintenance of Myc-driven lymphoma [4]. MRPL44 expression was identified as a predictor of lymph node metastasis in papillary thyroid carcinoma [5], and MRPL13 suppression was shown to be a key upstream regulator of OXPHOS dysfunction and hepatoma cell invasiveness [6]. In addition, mitoribosome defects due to collectively deregulated MRP expression was shown to promote an aggressive phenotype of hepatocellular carcinoma (HCC) by suppressing immune surveillance [7]. These results suggest that deregulation of MRPs may be responsible for bioenergetic dysregulation and crucially involved in tumor progression.

Somatic copy number alterations (SCNAs) are commonly found in many tumors and recognized as a key mechanism of cancer development through the activation of oncogenes and inactivation of tumor suppressors [8, 9]. SCNA analysis has often been employed to identify novel target genes for cancer progression, including HCC [8, 10, 11]. Because SCNAs affect a larger fraction of the genome in cancers than any other somatic genetic alteration [8], the involvement of unidentified individual genes needs to be elucidated within each SCNA region. From this perspective, unveiling the mechanistic link between SCNAs and mitoribosome deregulation may provide novel insights to elucidate the causative mechanism of bioenergetic dysregulation in cancer.

Collagens are the major structural components of the extracellular matrix (ECM) and form the scaffold of the tumor microenvironment, thereby regulating tumor infiltration, angiogenesis, and metastasis [12]. Collagen proteins are mainly produced and secreted by fibroblasts [13]. Recently, high expression of several collagens was found in diverse cancers [14]. Type I collagen is composed of three chains: two collagen type I alpha 1 (COL1A1) chains and one collagen type I alpha 2 (COL1A2) chain [15]. Aberrant COL1A1 expression has been implicated in some cancers, including HCC [16, 17], and demonstrated to be involved in hepatic fibrosis and HCC metastasis [18, 19]. However, how COL1A1 expression is regulated in hepatoma cells and how COL1A1 modulates the cells’ activities remain unclear.

In this study, we identified eight key SCNA-dependent MRPs by analyzing genomic and transcriptomic data from The Cancer Genome Atlas-Liver HCC database (TCGA-LIHC). Among them, *MRPS31* was the only gene linked to SCNA loss and poor prognosis. We demonstrate the involvement of *MRPS31* loss in mitoribosomal deregulation and subsequent OXPHOS defects, and in aggressive HCC phenotypes/activities. Together, our findings explain that *MRPS31* loss is a key driver of mitochondrial dysfunction and HCC aggressiveness by modulating hepatoma cell invasiveness, suggesting MRPS31 loss as a novel prognostic marker of HCC malignancy.

## Materials and methods

### Cell culture and cell growth rate

HepG2 and Hep3B cells were purchased from American Tissue Culture Collections (ATCC, Rockville, MD). SNU449 was obtained from Korean Cell Line Bank (Seoul, Korea) and JHH5 was from Japanese Collection of Research Bioresources Cell Bank (JCRB, Tokyo, Japan). HepG2, Hep3B, and SNU449 were cultured in RPMI1640 medium (Invitrogen, Carlsbad, CA) and JHH5 in William’s Media E (Invitrogen), respectively, supplemented with 10% fetal bovine serum (FBS) (Invitrogen) and antibiotics (Invitrogen) at 37 °C in a humidified incubator with 5% CO_2_. Cell lines were authentified by the short tandem repeats (STR) analysis (Cosmogenetech, Seoul, Korea). Cells were routinely tested for mycoplasma contamination by using e-Myco™ plus Mycoplasma PCR Detection Kit (iNtRON biotechology, Seongnam, Korea).

The cell growth rate was monitored by counting the trypan blue-negative viable cells. Cells seeded on 24 well plates were cultivated for 48 h, harvested by trypsinization, and then counted using the Countess™ automated cell counter (Invitrogen) after staining with 0.4% (w/v) trypan blue (Invitrogen) to exclude dead cells. In all experiments, no significant dead cells were observed.

### Preprocessing of public data

To interrogate the clinical significance of MRPs in HCC progression, transcriptome and DCN data HCC reposited in the TCGA-LIHC project were used. The level 3 RNA-seq v2.0 data of primary tumors (PT, *n* = 371)/nontumor surrounding tissue (NT, *n* = 50) and Affymetrix SNP 6.0 data of PT (*n* = 376)/paired NT (*n* = 376) were downloaded from the Genomic Data Commons Data Portal (https://portal.gdc.cancer.gov/projects/TCGA-LIHC) for transcriptome and DNA copy number (DCN) profiling, respectively. To match the features between transcriptome and DCN data, each gene of the segmented DCN data were mapped to the transcriptome features, resulting in total 58,387 matched genes. Among the 58,387 genes, 307 genes with DCN data of less than 50% of total HCC cases were excluded. As a result, 58,080 genes were used in further analysis. For all genes located in each segmented region, the same DCN values were assigned. To define deregulated expression (DE) in PT compared to NT, fragments per kilobase of transcript per million (FPKM) value for each annotated gene was generated for individual PT and normalized by its mean value of total NT. To define somatic copy number alteration (SCNA), each gene’s DCN value of PT were normalized by the mean of the matched gene’s values in NT. Finally. association between DE and SCNA was derived from the values of 369 HCC cases which harbored both transcriptome and DCN data by using Pearson’s product-moment correlation analysis.

For validation in HCC cell lines, we used RNA-seq gene expression data for the total of 28 HCC cell lines and DNA copy number data for 27 HCC cell lines from Cancer Cell Line Encyclopedia (CCLE). For transcriptome, Reads Per Kilobase of transcript per Million (RPKM) values were used to preprocess the expression value of each gene symbol. For DCN analysis, gene-level copy number data were used. For association analysis between mRNA and DCN, 27 HCC cell lines harboring both data were used. Correlation estimates and p value were calculated by applying Pearson’s product-moment correlation test. All processing was conducted using R packages of Bioconductor 4.1.0 (https://cran.r-project.org/doc/FAQ/R-FAQ.html).

### Identification of key SCNA- dependent MRPs

To identify key SCNA-dependent MRPs, we selected significantly up- (*n* = 19) or downregulated MRPs (*n* = 8) by performing permutated student’s *t* test between NT and PT [fold change (FC) > 0.3 or < −0.3 and permutated *p* value < 0.005]. Then, MRPs with DCN-gain (*n* = 12) or -loss (*n* = 5) were identified by SCNA analysis (FC > 0 or FC < 0). By employing Pearson’s product-moment correlation analysis of the two estimates, eight key SCNA-dependent MRPs were identified, showing concordantly dysregulated pattern in DE and SCNA (correlation estimate > 0.5, *p* value < 0.005).

### Calculation of SCNA frequency of high and low MRPS31-DCN groups

Based on the DCN of MRPS31, TCGA-HCC samples (*n* = 376) were stratified into the MRPS31_high (*n* = 94) or MRPS31_low (*n* = 94) group, respectively (upper or lower quartile of MRPS31-DCN). To define amplified (gain) or deleted (loss) status for 58,080 genes, cut-off threshold (>0.2 or < −0.2) was applied to the log2 transformed DCN value of each gene. In each group, the fraction of HCC samples for each genes’ status (gain or loss) was calculated, generating SCNA frequency, and plotted with their chromosome location. To distinguish DCN gain or loss, (-) value was assigned to the frequencies of genes with DCN-loss. Then, the differential SCNA frequency pattern was obtained by estimating differences of individual genes’ frequencies between the two groups (difference >0.2).

### HCC subclass prediction using the nearest template prediction (NTP) algorithm

Using the previously reported molecular subclass classifiers for HCC [Lee’s Poor prognosis [20], Woo_iCluster1 [21], TCGA_iCluster1 [22], Hoshida_subclass [23], Boyault_subclass [24] and Woo_CLHCC [25] (Suppl. Table S1)], a subclass for the individual sample of the MRPS31_high and _low groups was predicted by applying NTP algorithm with false discovery rate (FDR) < 0.05 [26]. Statistical significance was determined by Fisher’s exact test.

### Gene-set enrichment analysis (GSEA) and gene ontology (GO) analysis

To assess the enrichment of gene-sets of interest, we performed GSEA implemented in the software (http://software.broadinstitute.org/gsea/downloads.jsp) based on the Molecular Signatures Database (MSigDB database v7.0). Normalized enrichment score (NES) and FDR for each gene set were obtained. GO analysis was performed by using the gene sets assigned for the biological progress (BP) of MSigDB, V7.0 and the gProfileR of R package in *R* environment. The -log_10_(p value) values for the up- and downregulated genes were obtained.

### Estimation of genomic DCN

To isolate total genomic DNA, the conventional method was used. To estimate DCN, qPCR was performed using GoTaq® qPCR Master Mix (Promega, Fitchburg, WI, USA). Acidic ribosomal phosphoprotein P0 (34B4) gene, a known single copy gene [27], was used as an internal control. For each gene, two different DNA regions, which includes parts of the intron were selected. The primer sets were generated by Cosmogenetech (Seoul, Korea) and their sequences are as follows: MRPS31 Region 1 (5′-GTGTTCTTGGTTCATTTCGTG and 5′-GACTATCAAATAAATCTTGGGAG), MRPS31 Region 2 (5′-ATGCCTACAATCCTAGCTGC and 5′-CTCAGTTCGAACAGACTGC); RB Region 1 (5′-CATGTCAGAGAGAGAGCTTG and 5′-TAGCAGAGGTAAATTTCCTCTGG), RB Region 2 (5′-GATCTTTATTTTTTGTTCCCAGG and 5′GAACGACATCTCATCTAGGTC).

### Cellular RNA extraction and quantitative real-time PCR (qRT-PCR)

Total RNA was isolated using the NucleoSpin® RNA Plus kit (MACHEREY-NAGEL GmbH & Co. KG, Düren, Germany) and cDNA was prepared using ReverTra Ace™ qPCR RT Master Mix (Toyobo Co. Ltd., Osaka, Japan). qPCR was performed using GoTaq® qPCR Master Mix (Promega, Fitchburg, WI, USA). Expression levels of target mRNAs were normalized by β-actin or TBP mRNA level. The primer sets used were produced by Macrogen, Inc. (Seoul, Korea) and their sequences are as follows: MRPS31, 5′-GAAGAGCTGATCCAGTGGAC and 5′-CACAAGTCACCAGCTCCATG; RB1, 5′-CATGTCAGAGAGAGAGCTTG and 5′-GAACGACATCTCATCTAGGTC; MT-CO2, 5′-TGCCCTTTTCCTAACACTCAC and 5′-GGTTTGCTCCACAGATTTCAG; MT-ND6, 5-ATTGTTAGCGGTGTGGTCGG and 5′-CTCACCAAGACCTCAACCC; MRPS39, 5′-GACAGTCAGAAGCATTGGAAG and 5′-TCACCATTCCTCGGATCATTG; COL1A1, 5′-GCTCCAACGAGATCGAGATC and 5′-TACAGGAAGCAGACAGGGC; COL3A1, 5′-CCTGAAGCTGATGGGGTCAA and 5′-CCCCAGTGTGTTTCGTGCAA; MMP7, 5′-GCTACAGTGGGAACAGGCTC and 5′-GGGATCTCTTTGCCCCACAT; MMP14, 5′-CAAGATTGATGCTGCTCTCTTC and 5′-ACTTTGATGTTCTTGGGGTACT; LGALS4, 5′-CTACCAGCCCACCTACAAC and 5′-AGTGGAAGGCGACGTCTGAG; CA9, 5′-CTCGCTTGGAAGAAATCGCTG and 5′-CACTCAGCATCACTGTCTGGT; S100A11, 5′-CTCGCTCAGCTCCAACATG and 5′-AGGAACTCTGTCTTGGAGAG; CLDN4, 5′-AGCCTTCCAGGTCCTCAAC and 5′-GCGAGGTGACAATGTTGCT; ZEB1, 5′- CTACAACAACAAGACACTGCTG and 5′- TGTTCTTTCAGAGAGGTAAAGC; DDR1, 5′- CTCATCATTGCCCTCATGC and 5′- GCGGTTGTTGATGAGGATAG; β-actin 5′-CCTTCCTGGGCATGGAGTCCTGT, and 5′-GGAGCAATGATCTTGATCTTC.

### Western blot analysis

Western blotting was performed using standard procedures. Antibodies used are as follows: MRPS31 (ab167406), MT-COII (ab110258), MRPL13 (ab103801), MRPS15 (ab137070) and MRPL48 (ab194826) were purchased from Abcam (Cambridge, MA). α-tubulin (05-829) and VDAC (PC548) antibodies were obtained from Millipore (Billerica, MA) SDHA (MS204) antibodies were obtained from Mitoscience (Eugene, OR). Antibodies for RB (9309), HA (2367), SNAIL (3879), Slug (9585), ZEB1 (3396), and E-cadherin (3195) were obtained from Cell Signaling Technology (Danvers, MA). MRPS29 (GTX113864), COL1A1 (GTX112731), MMP7 (GTX104658), and DDR1 (GTX111453) antibodies were purchased from GeneTex Inc. (Irvine, CA). Antibodies against MT-ND6 (A31857) and MRPS39 (25158-1-AP) were purchased from Molecular Probes Corp. (Eugene, OR) and Proteintech Group, Inc. (Rosemont, IL), respectively. Antibodies for p21 (sc-6246) and p18 (sc-9965) were purchased from Santa Cruz (Dallas, TX). p16 antibody was purchased from BD bioscience (Franklin Lakes, NJ).

### Cell invasion assay

Cell invasion assay was performed with Transwell^TM^ Permeable Supports (8-µm pore size; Corning, Acton, MA) which was pre-coated with 7% Growth Factor Reduced BD Matrigel^TM^ Matrix (Becton Dickinson Labware, Franklin Lakes, NJ) as described previously [28]. All experiments were performed in at least three independent experiments.

### Transfection of siRNAs

To introduce cDNA plasmids and small interfering RNAs (siRNAs) into cells, cells were transfected with target siRNA duplexes using Oligofectamine^TM^ Reagent (Invitrogen), according to the instructions provided. Target siRNAs used in this study were obtained from Bioneer (Daejeon, Korea) and their sequences are as follows: MRPS31 siRNAs (#1, 5′-GAUUCUCUCCCUUUUGAUA; #2, 5′-CAGUUGUGUUCUUGGUUCA), MRPS39 siRNAs (#1, 5′-GAGAACUGAGAUAUACCAA; #2, 5′-GAACAAAAGGAAGCCCUAA), COL1A1 siRNAs (#1, 5′-CACCAAUCACCUGCGUACA; #2, 5′- UUGGUGUUGUGCGAUGACGUG), MMP7 siRNAs (#1, 5′-UGUUGCAGAAUACUCACUA, #2, 5′-CUCACUUCGAUGAGGAUGA), DDR1 siRNAs (#1, 5′-GGGACACCCUUUGCUGGUA; #2, 5′-GGGAUGGACUCCUGUCUUA), ZEB1 siRNAs (#1, 5′-CUGCUUUAUCCAUGUACUU; #2, 5′-CCUCUCUGAAAGAACACAUUA) and negative control siRNA (5′-CCUACGCCACCAAUUUCGU).

### Monitoring cellular oxygen consumption rate

To monitor mitochondrial respiratory activity, OCR was measured using Seahorse XF24 analyzer (Seahorse Bioscience Inc., MA) as described in the previous report [29].

### Construction and transfection of pcDNA3-MRPS31-HA vector

To generate pcDNA3-MRPS31-HA plasmid, conventional cloning procedures were applied. Briefly, MRPS31 cDNA fragment was amplified by PCR using total HepG2 cDNAs and a primer set, 5′-GCGAAGCTTATGTTTCCTAGAGTC and 5′-TACGCTCGAGTTAATTGAACTGTATG. The amplified MRPS31 cDNA was subcloned into HindIII and XhoI sites of the pcDNA3-HA vector.

To rescue the MRPS31 suppressed by siRNA, after MRPS31 had been knocked down by introducing siRNA for MRPS31 into cells using Oligofectamine^TM^ Reagent (Invitrogen) for 12 h, the cells were re-seeded and cultured for another 12 h and then transfected with the expression plasmid with Fugene® HD Reagent (Promega) for 2 days.

### Sucrose gradient sedimentation analysis for mitoribosome integrity

The sedimentation property of the mitoribosome complex was analyzed by sucrose gradient sedimentation as described previously with a slight modification [30]. Whole cells were solubilized in 500 μl lysis buffer [20 mM HEPES, pH 7.4, 100 mM KCl, 20 mM MgCl_2_, 1% triton X-100, 1 mM Na_3_VO_4_, 1 mM NaF, 1 mM PMSF, 1.5 μg/ml pepstatin A, 1.5 μg/ml leupeptin, 40U/ml recombinant RNase inhibitor (Promega, Madison, WI)]. After centrifugating the lysates at 13,000 rpm for 15 min at 4 °C to remove debris, the supernatant (200 μg) was loaded onto a 3 ml linear 10–40% sucrose gradient containing the mitochondrial lysis buffer and centrifuged at 40,000 rpm for 3 h 10 min at 4 °C using TLA-110 rotor and Beckman Optima MAX-TL (Beckman Coulter, Brea, CA). The linear gradients were collected from the bottom into 10 fractions, each fraction (40 μl) was subjected to Western blot analysis to identify individual MRPs.

### Monitoring mitochondrial translation activity

Mitochondrial translation activity was monitored using Click-iT^TM^ HPG Alexa Fluor^TM^ 488 Protein synthesis Assay Kit (Invitrogen, Carlsbad, CA) as described previously with a slight modification [31]. To label newly synthesized proteins at mitoribosome, cytosolic translation activity was inhibited by preincubating cells with 20 μg/ml emetine (Merck Millipore, Burlington, MA) for 1 h. Then, cells were incubated in methionine-free RPMI medium (Invitrogen) supplemented with 50 μM L-homopropargylglycine (Click-iT^TM^ HPG, Invitrogen) for 1 h. After washed twice with PBS, the cells were lysed in lysis buffer (1% SDS, 50 mM Tris, pH 8.0, 1 mM NaF, 1 mM Na_3_VO_4_, 1 mM PMSF, 1.5 μg/ml pepstatin A, 1.5 μg/ml leupeptin). Whole-cell lysate (50 μg) was further conjugated with Alexa Fluor 488 in a Click-iT^TM^ reaction cocktail for 30 min according to the manufactures protocol and subjected to Western blot analysis using Alexa Fluor 488 antibody (A-11094, Invitrogen). The specificity of mitochondrial translation activity was confirmed using chloramphenicol (Sigma, St. Louis, MO).

## Results

### MRPS31 is the key SCNA-dependent MRP suppressed in HCC

**Fig. 1:**
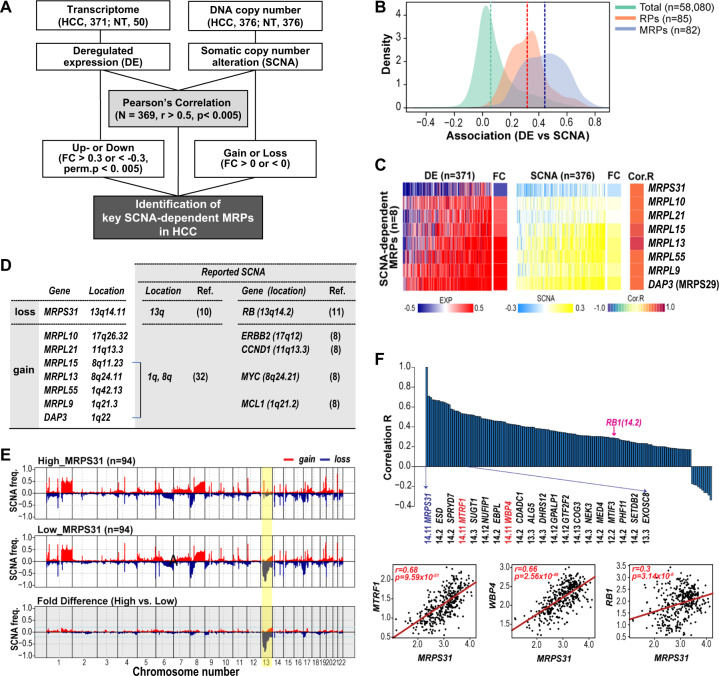
Identification of key SCNA-dependent MRPs in HCC. **A** Schematic flow of the analysis to identify key SCNA-dependent MRPs. **B** Histograms of the association between DE and SCNA of total genes, RPs, and MRPs. **C** Heatmaps of DE (left), SCNA (middle), and Cor. R (right) of eight SCNA-dependent MRPs. Average fold changes (FC) of the HCC samples’ DE and SCNA values are included. **D** Commonly reported SCNA-associated locations and genes and their link to the MRPs. **E** Whole chromosome SCNA frequencies of high and low MRPS31-DCN groups and their fold differences (bottom). Chromosome boundaries (perpendicular solid lines) and centromere positions (dashed) are indicated and chromosomal region with substantial frequency differences between the two groups was colored yellow. **F** Correlation estimates between expressions of MRPS31 and the genes located in the yellow-colored region of (**E**). The top 20 genes with high correlation are listed with their location. Scatter plots (lower panel) of the association between expressions of MRPS31 and neighboring genes (MTRF1 and WBP4) or RB1. The statistical r and p values by Pearson’s product-moment correlation test are depicted.

Previously, we reported that deregulation of MRPs primes a favorable cancer microenvironment, promoting HCC progression [7]. In this study, we hypothesized that SCNA is a causative mechanism of mitoribosomal deregulation, and SCNA-dependent MRPs are key driver genes in HCC. We performed genomic and transcriptomic profiling of 82 MRPs using both the DCN and transcriptome data of TCGA-LIHC cohorts (Fig. 1A). First, we derived the deregulated expression (DE) and SCNAs of each MRP in individual HCC samples. Then, we interrogated the association between the SCNA and DE values for the MRPs by performing Pearson’s correlation analysis. Global MRPs (*n* = 82) had higher correlations (median > 0.4) than total transcripts (*n* = 58,080) or cytosolic ribosomal proteins (RPs; *n* = 85; Fig. 1B). This suggests that SCNA is primarily involved in modulating MRP expression in HCC.

Next, we determined eight key SCNA-dependent MRPs [*MRPS31*, *MRPL10*, *MRPL21*, *MRPL15*, *MRPL13*, *MRPL55*, and *DAP3* (MRPS29)] that had higher correlation estimates (Cor. R; *P* < 0.005, *r* > 0.5; Fig. 1C, Suppl. Fig. S1). Interestingly, the chromosome locations of the eight MRPs were closely related to the commonly reported SCNA-associated locations and/or genes (Fig. 1D) in HCC [10, 11, 32] and pan-cancer [8]. *MRPS31* (13q14.11) is located within the 13q locus, which is reported to be the recurrently deleted locus in HCC [10] and resides close to *RB1* (13q14.2) [11]. *MRPL10* and *MRPL21* are situated near the oncogenes ERBB2 and CCND1, respectively, which have been reported to be gene amplifications [8]. *MRPL15* and *MRPL13* reside within 1q, and *MRPL55*, *MRPL9*, and *DAP3* within 8q, which have been identified as hot spots for DCN gain in HCC [32]. These findings indicate that the SCNAs of the eight MRPs may occur simultaneously with common SCNA events during HCC development.

Among the eight MRPs, we focused on *MRPS31* because it was the only MRP showing downregulation with DCN loss. Therefore, we hypothesized that MRPS31 loss is the key mechanism underlying mitoribosomal deficiency and subsequent defects in mitochondrial bioenergetics. When we compared the SCNA frequencies on whole chromosomes between the two groups (*n* = 94 each) with high- and low MRPS31 DCNs, we observed a remarkable difference in the 13q14.11-14.3 locus (Fig. 1E). This result revealed that MRPS31 loss was tightly linked to the collective deletion of the 13q14.11-14.3 locus and was independent of the other SCNA events, such as amplification of the other seven MRPs and common gain of the 1q and 8q locus. This was further supported by the finding that the expression of most of the genes in this region was highly associated with MRPS31 (Fig. 1F and Suppl. Fig. S2). Among the genes, *MTRF1* (13q14.11) and *WBP4* (13q14.11) neighboring *MRPS31* had a high positive association (*r* > 0.66) with *MRPS31* expression, and *RB1* (13q14.2) also showed positive association (*r* = 0.3; Fig. 1F and Suppl. Fig. S2).

### MRP31 loss is closely associated with poor prognosis and aggressive HCC phenotypes

**Fig. 2:**
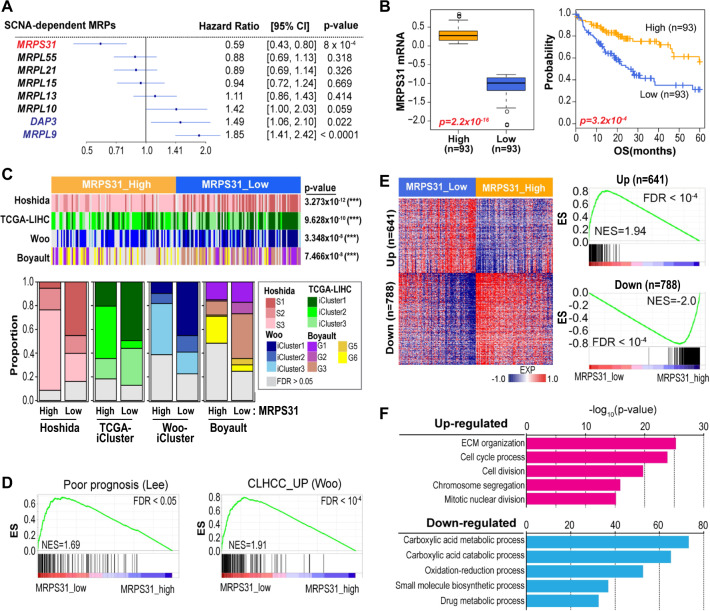
MRPS31 loss is closely associated with poor clinical outcome of HCC. **A** Forrest plot of the hazard ratios for the key SCNA-dependent MRPs by the univariate Cox-regression survival analysis. Their confidence interval (CI, 95%) and *p* values are indicated. **B** Boxplot of MRPS31 mRNA levels (left) and overall survival times (OS) using the KM survival analysis (right). Based on MRPS31 mRNA level, TCGA-LIHC samples were stratified into MRPS31_high (*n* = 93) or MRPS31_low (*n* = 93) groups (upper or lower quartile). **C** KM survival curve. Based on *RB1* DCN (threshold of −0.2 for DCN loss), TCGA-LIHC samples were divided into RB1 wild type (*n* = 210) and RB1 deleted type (*n* = 93) group and then the RB1 wild type group was subdivided into MRPS31_high (*n* = 52) and MRPS31_low (*n* = 52) groups (upper and lower quartile, respectively). The statistical *p* value by the Cox-Mantel log-rank test was depicted for **(B)** and **(C)**. **D** Heatmap (top) of HCC classifier expressions and proportions of the predicted classifiers (bottom) obtained by performing NTP algorithm for the high and low MRPS31 groups. The statistical *p* values by Fisher’s exact test are noted. **E** Enrichment score (ES) by gene-set enrichment analysis (GSEA) using the Lee’s Poor prognosis A (left) and Woo’s CLHCC_UP (right) gene-sets. Normalized enrichment score (NES) and false discovery rate (FDR) for each gene set are noted. **(F)** Expression heatmap (left) and ES plots (right) of up (*n* = 641) or downregulated (*n* = 788) genes in the high and low MRPS31 groups. NES and FDR for each gene set are indicated. **G** Barplots of GO analysis using the biological process (BP) gene sets.

Given the close association of *MRPS31* with *RB1*, a core tumor suppressor, we questioned whether *MRPS31* loss could substantially contribute to HCC development rather than be an epiphenomenon. Thus, we investigated the relationship between the eight SCNA-dependent MRPs and clinical outcomes using the Cox-regression hazard ratio model. *MRPS31* expression had the lowest hazard ratio, meaning that *MRPS31* loss was closely associated with poor prognosis, whereas MRPS29 (*DAP3*) and *MRPL9* had high hazard ratios (Fig. 2A). After stratifying HCC samples into MRPS31_high (*n* = 93) and MRPS31_low (*n* = 93; > upper and < lower quartile, respectively), we performed Kaplan-Meier (KM) survival analysis and found that the MRPS31_low group had significantly shorter overall survival (Fig. 2B), implying a potential contribution of *MRPS31* loss to the clinical outcome of HCC. Although *MRPS31* showed a positive association with *RB1* at mRNA level (Fig. 1F), to further examine the relationship between *MRPS31* and *RB1* loss in the clinical outcome of HCC, we divided the HCC samples into RB1 wild type (*n* = 210) and RB1 deleted type (*n* = 166) groups, based on *RB1*-DCN (threshold of −0.2 for DCN loss). Then, we again stratified the RB1 wild type group into MRPS31_high (*n* = 52) and MRPS31_low (*n* = 52; > upper and < lower quartile, respectively) expression subgroups and performed KM survival analysis. Interestingly, the MRPS31_low subgroup of the RB1 wild type group showed significantly shorter overall survival (Fig. 2C). This result suggests that *MRPS31* loss may serve as a poor survival marker regardless of *RB1* loss.

To interrogate the molecular features closely related to MRPS31, we performed HCC subclass prediction using the nearest template prediction (NTP) algorithm [26] based on the reported HCC classifiers (Suppl. Table S1). Interestingly, the MRPS31_low group was significantly enriched with aggressive phenotype classifiers, including Hoshida_S1 [23], TCGA_iCluster1 [22], Woo_iCluster1 [21], and Boyault_G3 [24] (Fig. 2D). Moreover, gene signatures for Lee et al.’s poor prognosis [20] and cholangiocarcinoma-like HCC (CLHCC) [25] were more significantly enriched in the MRPS31_low group (Fig. 2E, Suppl. Table S1). We also identified significantly upregulated (*n* = 641) and downregulated genes (*n* = 788) in the MRPS31_low group by comparing the high and low groups (permutated student *t* test, *P* < 0.001; fold difference > 0.5; Fig. 2F). Gene Ontology (GO) analysis revealed that genes involved in ECM organization, mitotic cell division, and chromosome segregation were upregulated in the MRPS31_low group, whereas genes linked to the metabolic process were downregulated (Fig. 2G). Taken together, these results indicate that *MRPS31* loss is closely associated with poor prognosis and aggressive HCC phenotypes. Surprisingly, by analyzing the transcriptome data of 17 different cancer types in TCGA, we found that *MRPS31* is down-expressed in many tumor tissues (70%, 12/17) compared to their nontumor tissues (Suppl. Fig. S3; Suppl. Table S2). However, the clinical significance of MRPS31 suppression based on analyses of both the KM survival curve and Cox-regression hazard ratio was only found in HCC (Suppl. Fig. S4; Suppl. Table S2), emphasizing the unique dependency of HCC on MRPS31 downregulation.

### SCNA-dependent MRP31 suppression is linked to hepatoma cell invasiveness

**Fig. 3:**
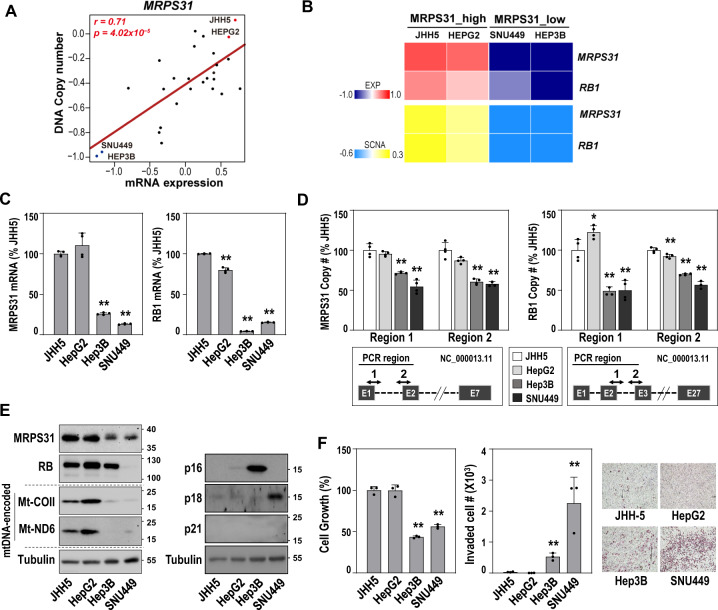
Hepatoma cells with MRPS31 loss displays high cell invasion activity. **A** Scatter plot of the association between mRNA level and DCN of MRPS31 of the 27 HCC cell lines. The statistical r and p values by Pearson’s product-moment correlation test are noted. Cell lines with top two highest (red) and two lowest (blue) MRPS31 levels are indicated. **B** Heatmap of mRNA levels and DCN of MRPS31 and RB1. **C** qRT-PCR quantification of four independent experiments. **D** DCN of MRPS31 (left) and RB (right) by performing genomic PCR against two different DNA regions (lower panels) for each gene (*N* = 4 for quantification). **E** Western blots. Protein expressions of two mtDNA-encoded genes, COII (Mt-COII) and ND6 (Mt-ND6) were included. **F** Cell growth (left) and cell invasiveness (middle). Representative images of invaded cells (right) are shown (*N* = 3 for quantification). **p < 0.01; *p < 0.05 vs. JHH5 by unpaired two-way student *t* test.

To select appropriate cell lines for elucidating the role of MRPS31 loss in hepatoma cell activities, we analyzed the transcriptome and DCN data for liver cancer cell lines in the Cancer Cell Line Encyclopedia (CCLE) (Suppl. Table S3). Analogous to the results from TCGA-LIHC (Fig. 1C and 1F), we found a significantly high association (*n* = 27; *r* = 0.71, and *P* < 0.001) between the mRNA expression and DCN of MRPS31 (Fig. 3A). Next, we decided to use two MRPS31_high type cells (HepG2 and JHH5) and two MRPS31_low type cells (Hep3B and SNU449). In these four hepatoma cell lines, DCN and mRNA expressions of *RB1* exhibited a similar pattern as *MRPS31* (Fig. 3B), which was expected from the TCGA-LIHC analysis. The mRNA levels, DCNs, and protein expression of MRPS31 and RB1 were validated (Fig. 3C–E). The two MRPS31_low type cells also had low protein expression of mtDNA-encoded *COII* and *ND6*, indicating the mitoribosomal deficiency of the cells (Fig. 3E, left). Moreover, the MRPS31_low type cells presented high cellular invasion activity despite their delayed cell growth (Fig. 3F). These results suggest that MRPS31 suppression is dependent on the SCNA loss of the chromosome 13q14 region in hepatoma cells and is closely associated with invasiveness. The two MRPS31_low type cells harbored increased expression of cyclin-dependent kinase inhibitors, such as p16^INK4A^ or p18^INK4C^ (Fig. 3E, right), suggesting their involvement in the delayed growth of the cells with *RB*1 loss. It is noteworthy that expressions of 35 MRPs were decreased in the MRPS31_low type cells (Suppl. Fig. S5), implying that *MRPS31* loss is not the only event for their mitoribosome defect.

### MRP31 suppression is a key mechanism of mitochondrial defects and hepatoma cell invasiveness

**Fig. 4:**
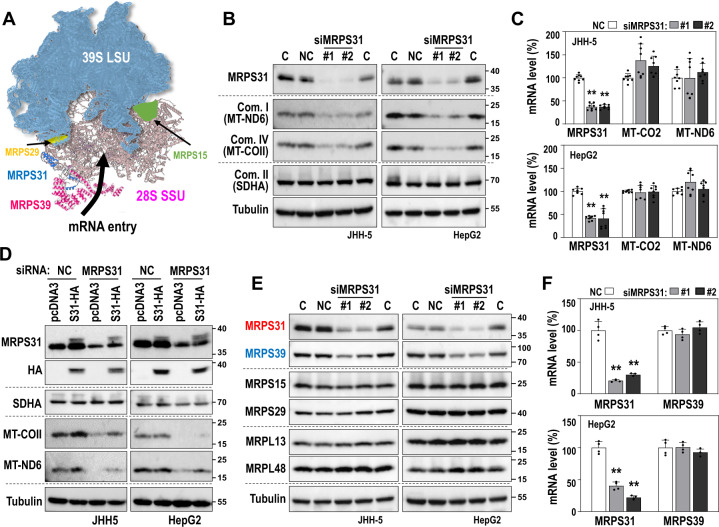
MRPS31 protein level is linked to MRPS39 protein level at translation level. **A** The schematic structure of human mitoribosome based on protein data bank (PDB). The 39 S large subunits (LSU) is colored in light blue and 28 S small subunits (SSU) in pink. Some small subunit proteins (MRPS29, MRPS31, MRPS39 and MRPS15) were marked with different colors. The mRNA entrance channel is located nearby MRPS39. **B–F** JHH5 and HepG2 cells were transfected with MRPS31 siRNA in the absence or presence of pcDNA3-MRPS31-HA plasmid for 3 days. **B, D, E** Western blot analysis. **C, F** qRT-PCR. Quantification of eight independent experiments for (C) and four experiments for (**F**). **p < 0.01 vs. siNC.

In the mitoribosome, MRPS31 is structurally attached to MRPS39 (Fig. 4A), which takes part in mitochondrial mRNA entry [33]. However, MRPS31 is located outside of mitoribosome without any RNA interactions [34]. Therefore, whether MRPS31 is an essential component to maintaining mitoribosomal activity needs to be proven. When we knocked down MRPS31 in HepG2 and JHH5 cells (MRPS31_high type), the protein levels of mtDNA-encoded genes *ND6* and *COII* were effectively suppressed without significant changes in their mRNA levels (Fig. 4B, C) or the ncDNA-encoded SDHA protein level (Fig. 4B). The addition of MRPS31 could restore the suppressed ND6 and COII protein levels (Fig. 4D), showing its specific action on the mitoribosome. Interestingly, MRPS31 knockdown also decreased the protein level of MRPS39, an interacting neighbor, without altering its mRNA level (Fig. 4E, F). However, it did not affect the other small (MRPS29 and MRPS15) and large (MRPL13 and MRPL48) subunit proteins. These results imply that MRPS31 loss frustrates MRPS39 protein integrity, thereby manifesting or amplifying the effect of MRPS31 loss on the mitoribosome activity.

**Fig. 5:**
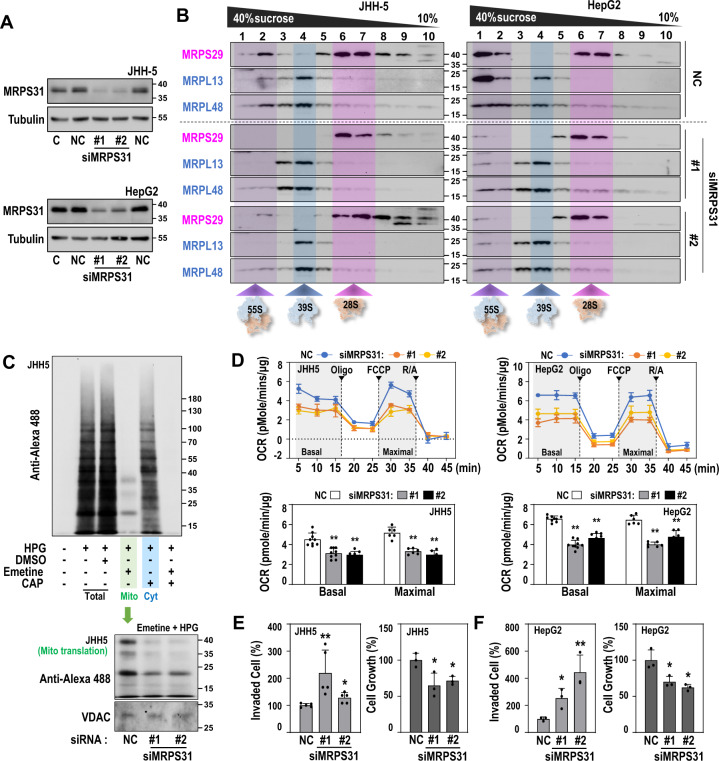
MRPS31 suppression induces mitoribosomal dysfunction, OXPHOS defect, and cell invasiveness. MRPS31_high type cells (JHH4 and HepG2) were transfected with MRPS31 siRNA for 3 days. **A** Western blots. **B** Western blots after sucrose gradient sedimentation analysis of mitoribosome. MRPS29 (a small subunit protein) and MRPL13 and MRPL48 (large subunit proteins) were used as indicators of each subunit. Purple, blue and pink colored box indicated 55 S monosome, 39 S large subunit, and 28 S small subunit, respectively. **C** Mitochondrial translation activity. *De novo* synthesized proteins using L-homopropargylglycine (HPG)-conjugated Alexa Fluor 488 were detected by Western blot analysis. To distinguish cytosolic and mitochondrial translation activities, emetine (a cytosolic translation inhibitor) and chloramphenicol (CAP, a mitochondrial translation inhibitor) were used. The effect of MRPS31 knockdown on mitochondrial translation is shown in bottom panel. **D** Cellular OCR of JHH5 (left) and HepG2 (right) and their quantifications (basal and maximal rates, bottom). Nine independent experiments were performed. **p < 0.01 vs. siNC by student *t* test. **E, F** Cell invasion activity (left, *N* = 5) and cell growth (right, *N* = 3) of MRPS31-knockdowned JHH5 **(E)** and cell invasion activity (left, *N* = 3) and cell growth (right, *N* = 3) of MRPS31-knockdowned HepG2 **(F)**. *p < 0.05 and **p < 0.01 vs. siNC.

The functional mitoribosome complex, a 55 S whole complex, is formed by joining a 39 S large subunit (LSU) and a 28 S small subunit (SSU) after biogenesis of each subunit is completed via independent pathways [34, 35]. The status of mitoribosome subunit assembly can be estimated by using sucrose gradient sedimentation analysis [30]. So, we examined the effect of MRPS31 knockdown on mitoribosome assembly using the method. In both HepG2 and JHH5 cells, MRPS31 knockdown decreased the formation of the 55 S whole ribosome complex (Fig. 5A, B) and mitoribosomal translation activity (Fig. 5C). Consequently, MRPS31 knockdown significantly decreased the mitochondrial oxygen consumption rate, both basal rate and maximum capacity (Fig. 5D). Furthermore, MRPS31 knockdown increased the cell invasion activity despite its suppressive effect on cell growth (Fig. 5E, F). These results indicate that MRPS31 is an essential component to maintaining mitoribosome activity, and its loss is a key mechanism in regulating hepatoma cell invasiveness.

### MMP7 and COL1A1 are downstream effectors of *MRPS31* loss on hepatoma cell invasiveness

**Fig. 6:**
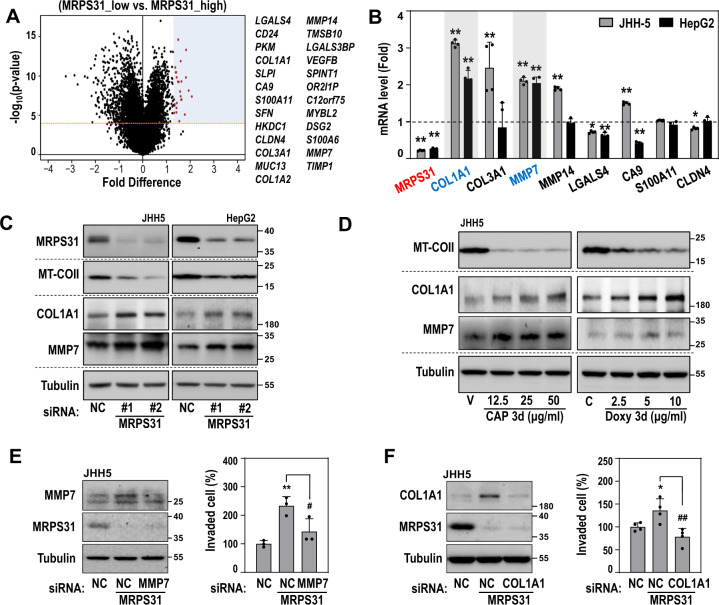
MMP7 and COL1A1 are downstream effectors of MRPS31 loss to enhance hepatoma cell invasiveness. **A** Volcano plot of total transcripts (*n* = 58,387) in TCGA-LIHC, using fold difference and *p* values of the expression level, based on the permutated student *t* test between the MRPS31_high (*n* = 93) or _low (*n* = 93) group. Names of highly upregulated genes (fold difference > 1.5 and permutated *p* < 10^−4^, blue region) are listed (right). **B, C** MRPS31_high type cells (JHH4 and HepG2) were transfected with MRPS31 siRNA for 3 days. **B** Messenger RNA levels by qRT-PCR. Fold changes against siNC are displayed (*N* = 4). *p < 0.05 and **p < 0.01 vs. siNC by student *t* test. **C** Western blots. **D** Western blots of JHH5 cell exposed to the mitochondrial translation inhibitors, chloramphenicol (CAP) and doxycycline (Doxy), for 3 days. **E, F** JHH5 cells were transfected with MRPS31 siRNA in the absence or presence of siMMP7 (*N* = 3) **(E)** or siCOL1A1 (*N* = 4) **(F)** for 3 days. Western blots (left) and cell invasiveness (right). *p < 0.05 and **p < 0.01 vs. siNC; #*p* < 0.05, and ##*p* < 0.01 vs. siMRPS31.

To interrogate the downstream effector(s) of MRPS31 loss, we compared gene expression between the MRPS31_high and MRPS31_low groups by performing a permutated student *t* test and selecting the 25 top-ranked genes from the significantly upregulated genes in MRPS31_low (*P* < 10^−4^ and fold difference > 1.0; Fig. 6A). Next, we screened potential downstream targets of MRPS31. COL1A1 and MMP7 mRNA levels were commonly upregulated by MRPS31 knockdown in both JHH5 and HepG2 cells (Fig. 6B and Suppl. Fig. S6A). In addition, MRPS31 knockdown augmented the COL1A1 and MMP7 protein levels (Fig. 6C). Inhibition of mitoribosomal translation activity by mitoribosome-specific inhibitors chloramphenicol and doxycycline also increased COL1A1 and MMP7 protein levels (Fig. 6D). However, among the two MRPS31_low type cells (Hep3B and SNU449), only SNU449 harbored high COL1A1 and MMP7 expression (Suppl. Fig. S6B), suggesting that Hep3B may possess other suppressive mechanism(s). Individual knockdown of MMP7 and COL1A1 significantly decreased the invasiveness of SNU449 cells (Suppl. Fig. S6C, D). In addition, knockdown of either COL1A1 or MMP7 along with MRPS31 knockdown partially, but significantly, attenuated the MRPS31 depletion-mediated invasiveness (Fig. 6E, F), indicating that the invasiveness acquired by MRPS31 suppression is mediated through MMP7 and COL1A1 expression.

### COL1A1 is a key player in hepatoma cell invasiveness via ZEB1-mediated EMT

**Fig. 7:**
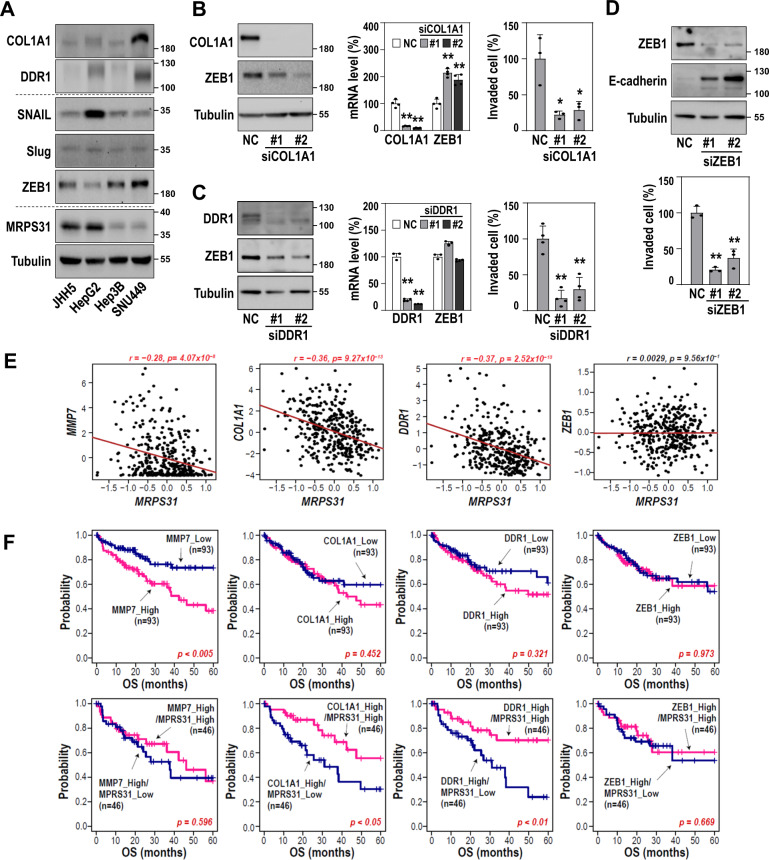
COL1A1 is a key player of liver cancer cell invasiveness via ZEB1-mediated EMT. **A** Western blots. **B–D** SNU449 cells were transfected with siRNAs against COL1A1 **(B)**, DDR1 **(C)**, and ZEB1 **(D)**. **B, C** Western blots (left), qRT-PCR (middle, *N* = 4), and cell invasiveness [right, *N* = 3 for (**B**) and *N* = 4 for (**C**)]. *p < 0.05 and **p < 0.01 vs. siNC. **D** Western blots (top) and cell invasiveness (bottom, *N* = 3). **p < 0.01 vs. siNC. **E** Scatter plots of the associations of MRPS31 expression with the indicated genes. The statistical r and p values by Pearson’s product-moment correlation test are depicted. **F** KM curves with OS. TCGA-LIHC samples were stratified into high or low expression group (upper or lower quartile) of the indicated genes (top). The high expression groups of the individual genes were further stratified into MRPS31 high or low expression group by using median values (bottom). The statistical p values by Cox-Mantel log-rank test are depicted.

We also examined how hepatoma cell-derived COL1A1 regulates invasiveness. SNU449 cells possessed high protein levels of DDR1, a cellular receptor of collagen [36], and ZEB1, a key transcriptional regulator of EMT (Fig. 7A). Individual knockdown of COL1A1 and DDR1 effectively decreased the protein levels of ZEB1 without diminishing the mRNA level (Fig. 7B, C), which is supported by a report that ZEB1 is regulated at the protein level in HCC [37]. In addition to COL1A1 and DDR1 knockdown, ZEB1 suppression significantly decreased SNU449 cell invasiveness (Fig. 7B–D), implying involvement of the COL1A1/DDR1/ZEB1 axis in hepatoma cell invasiveness.

By analyzing the transcriptome in TCGA-LIHC, we observed that MRPS31 is negatively associated with MMP7, COL1A1, and DDR1, but not with ZEB1 (Fig. 7E). Furthermore, KM survival analysis revealed that only the MMP7 level could stratify the prognosis of HCC patients using overall survival time (upper panels of Fig. 7F), suggesting a potential prognostic use of MMP7. However, either the COL1A1_high or DDR1_high group (n = 93) could be clearly stratified by MRPS31 level, showing worse prognosis with the MRPS31_low group (*n* = 46) (bottom panels of Fig. 7F). These results show that *MRPS31* loss-mediated COL1A1/DDR1 can predict HCC prognosis. Overall, our data suggest that *MRPS31* loss may be a key regulator of HCC and a novel biomarker for clinical application.

## Discussion

Although mitoribosomal activity governs the protein translation of only 13 mtDNA-encoded proteins, it is critical to maintain mitochondrial bioenergetics because these 13 proteins are core components of the OXPHOS system. Therefore, deregulation of the MRPs comprising the mitoribosome undoubtedly affects mitoribosomal functional integrity, contributing to ‘deregulated cellular bioenergetics,’ a key hallmark of cancer [38]. Previously, we reported that global MRP deregulation promotes an aggressive phenotype of HCC [7]. However, its causative mechanism is not fully understood. In this study, we hypothesized that SCNA, a key mechanism in cancer development [8, 9], may have a causative role in MRP deregulation. We found that MRP expression patterns are tightly associated with their SCNA in HCC, implying the potential contribution of SCNA to deregulation. We further identified eight key SCNA-dependent MRPs. Strikingly, the chromosomal locations of all of these MRPs were closed linked to the commonly reported SCNAs in HCC, suggesting that the MRP genes are located in the same vulnerable chromosome regions as SCNAs. This is an important novel finding, suggesting direct SCNA-driven mitochondrial dysregulation in cancer.

Among the eight MRPs, only MRPS31 exhibited SCNA loss-mediated suppression. Interestingly, *MRPS31* (13q14.11) is located close to *RB1* (13q14.2) within chromosome 13q, which has been frequently found with DCN loss [10, 11], loss of imprinting [39], and loss of heterozygosity [40] in HCC. The association between *MRPS31* loss and *RB1* loss was estimated to be high, indicating that it is a consequence of a simultaneous deletion event. Moreover, Jee et al.’s SCNA analysis of stepwise HCC samples, such as liver cirrhosis, low-grade dysplastic nodule, early HCC, and progressed HCC, revealed that prominent SCNA loss in the 13q region is present in the early stages of HCC [10]. This suggests that *MRPS31* loss and the associated mitochondrial dysfunction may also occur in an early stage of cancer. Considering the key tumor-suppressive role of RB, the dependence of *MRPS31* loss on *RB1* deletion prompted us to ask whether *MRPS31* loss is just an epiphenomenon during HCC development. MRPS31 downregulation alone showed a close association with poor prognosis and aggressive HCC phenotypes. Notably, the dependency of the clinical outcome on MRPS31 expression was a unique feature of HCC, implying a possible stronger dependence of hepatoma cell activities on mitochondrial dysfunction. However, the clinical outcome of MRPS31 downregulation may still be inferred as the result of coincident *RB1* deletion. We proved that MRPS31 suppression is enough to induce mitoribosomal defects and subsequent mitochondrial dysfunction, eventually triggering hepatoma cell invasiveness. These results explain that MRPS31 is an essential component for mitoribosomal activity, and its SCNA-dependent downregulation is a key regulatory mechanism of hepatoma cell invasiveness and HCC malignancy. In addition, *MTRF1* (13q14.11), the gene for mitochondrial translation release factor 1 that functions in the termination of mitochondrial translation, was also coincidently deleted with MRPS31, implying its additional contribution to mitoribosomal defects.

In the past few decades, altered MRP expression has been implicated in the progression and metastasis of many cancer types. For example, increased MRPS23 expression contributes to HCC cell proliferation and poor clinical outcome [41], and its gene amplification has been observed in cervical and breast cancer [42]. In addition, MRPS5 enhances the metabolic flexibility of liver cancer stem cells [43]. In contrast, decreased MRPL11 is considered a potential biomarker of primary head and neck squamous cell carcinoma [44]. However, MRPL13 suppression is a cause of mitochondrial defects in HCC and promotes the invasiveness of mitochondria-defective hepatoma cells. Furthermore, a few alternative roles of several MRPs, such as MRPS29/*DAP3* [45], *MRPS30/PDCD9* [46], and *MRPL41* [47], have been reported in the induction of apoptosis. Recently, a critical role of MRPS39, also called PTCD3, was demonstrated in Myc-driven lymphoma [4]. MRPS39, which resides outside the projecting part of the ribosome, takes part in mitochondrial mRNA entry; it directly interacts with mitochondrial mRNAs to proceed with their translation, implying a crucial role in mitoribosomal activity [33, 48]. On the other hand, MRPS31 is closely attached to MRPS39 in the exterior of the mitoribosome without any RNA interaction [34]. In this study, we showed that MRPS31 has an interdependent relationship with MRPS39 at the protein level, and that MRPS31 suppression attenuates the assembly of the whole 55 S mitoribosome and mitoribosomal translation activity, eventually decreasing the mitochondrial OXPHOS activity. These findings suggest that MRPS31 is an essential component for functional mitoribosome assembly, together with a role in maintaining MRPS39 protein integrity. This finding is further supported by a recent study on mitochondrial ribosome assembly, which classified MRPS31 as a core early assembly component [34].

At the present time, it is not clear how *MRPS31* loss leads to increased expression of COL1A1 and MMP7. One possible explanation can be made based on our previous report that mitoribosomal defect augments *TGFB1* expression and TGF-β-mediated responses [7]. TGF-β1 is known to regulate MMP7 via Smad signaling or the β-catenin pathway [49]. In addition, Pan X. et al. reported that TGF-β1 induced collagen type I expression through DNMT-mediated DNA demethylation of the COL1A1 promoter [50]. These findings present a potential role of TGF-β1 as a mediator in the *MRPS31* loss-derived MMP7 and COL1A1 expression. In an effort to elucidate how MRPS31 loss-mediated mitoribosomal defects modulate hepatoma cell activity, we identified COL1A1, a component of type I collagen, as a key downstream effector molecule. As a major component of the ECM, collagen modulates cancer cell metastasis by stiffening the ECM structure [51]. Rapid migration of breast cancer cells and leukocytes along the collagen fiber was visualized [52] and a role of remodeled stiff collagens in invasion was demonstrated in glioma cells [53]. Recently, COL1A1 was identified as a reliable biomarker of HCC metastasis [18]. However, collagen proteins, including COL1A1, are mainly produced and secreted by fibroblasts [13]. In this study, we demonstrated that COL1A1 is produced by hepatoma cells with MRPS31 loss, and COL1A1/DDR signaling could enhance cell invasiveness by activating the ZEB1-mediated EMT, thereby contributing to HCC aggressiveness. These results suggest that a combination of MRPS31 loss and an upregulated COL1A1/DDR axis can be used as an effective diagnostic marker in HCC.

## Supplementary information

Supplementary Figure S1-S6

Supplementary Table S1-S3

## Data Availability

The datasets analyzed during the current study are available from the corresponding author on reasonable request.
