# Supplementary material for: MRPS31 loss is a key driver of mitochondrial deregulation and hepatocellular carcinoma aggressiveness
Source: Cell Death Dis. 2021 Nov 12;12(11):1076. doi: 10.1038/s41419-021-04370-8 (PMC8589861; doi:10.1038/s41419-021-04370-8)
Supplement: Supplementary file 1 — Supplementary Figure S1-S6 [file 41419_2021_4370_MOESM1_ESM.pdf]

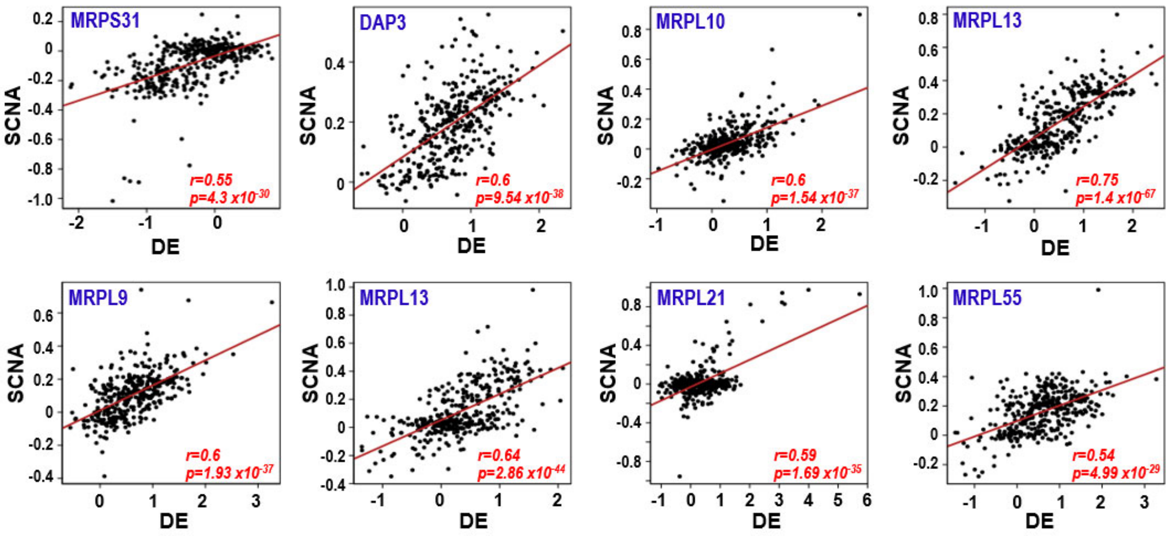

Suppl. Figure S1. Identification of SCNA dependent MRPs in TCGA-LIHC.
Scatter plots indicate the association between DE and SCNA of the eight selected SCNA-dependent MRPs in TCGA-LIHC. Correlation estimates and p-values based on Pearson's product-moment correlation test are depicted.

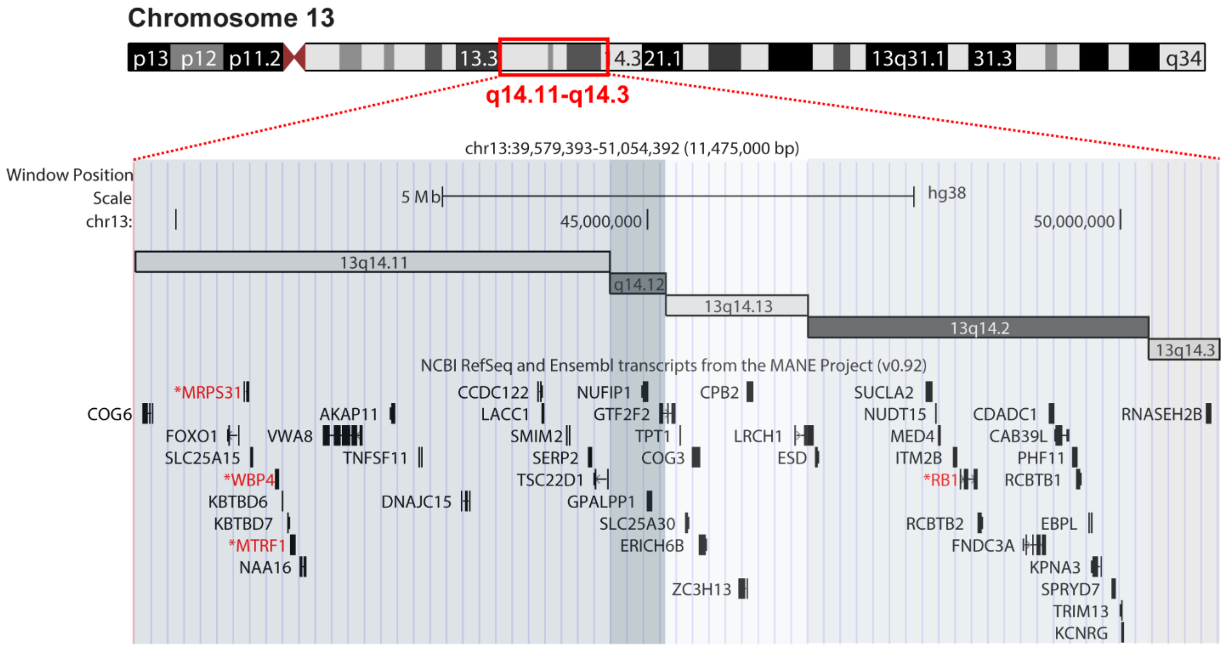

Suppl. Figure S2. Genomic region deleted in MRPS31_low DCN group.

Ideogram of chromosome 13, focusing the cytobands (q14.11-q14.3). Among the genes located in the genomic region of 13q14.11 to 13q14.3, the positions and names of the genes correlated with MRPS31 expression are listed. Red colored genes were individually analyzed for association with MRPS31 using scatter plot in Fig. 1F.

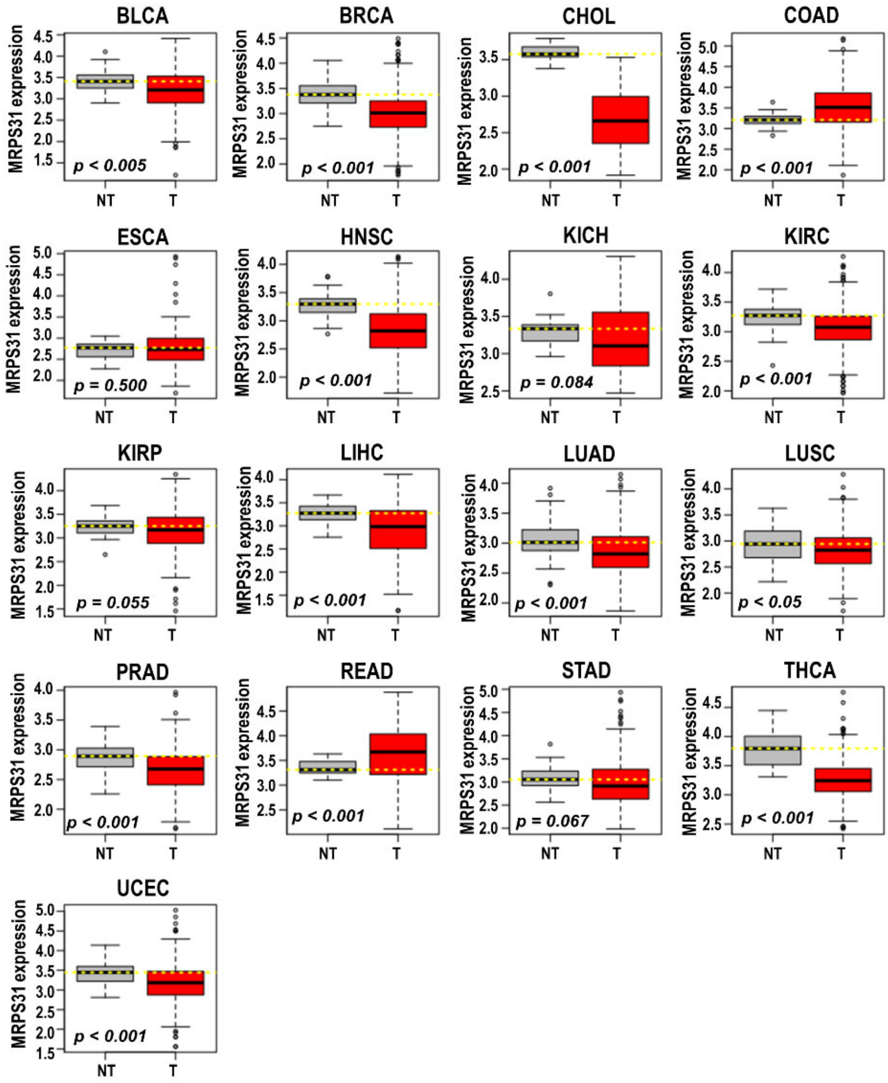

Suppl. Figure S3. Comparison of MRPS31 expression between tumor and non-tumor tissues of 17 different cancer types using TCGA transcriptome data.

Boxplots indicate the MRSP31 expression levels of non-tumor (NT) and tumor (T) tissues in different type cancers. For validation in pan-cancer cohorts, transcriptome data of seventeen different type cancer cohorts with more than five non-tumor tissues reposited in TCGA were used to compare MRPS31 expression levels between non-tumor and tumor tissue. The cancer cohorts used in this study as follows: bladder urothelial carcinoma (BLCA), breast invasive carcinoma (BRACA), cholangiocarcinoma (CHOL), colon adenocarcinoma (COAD), esophageal carcinoma (ESCA), head and neck squamous cell carcinoma (HNSC), kidney chromophobe (KICH), kidney renal clear cell carcinoma (KIRC), kidney renal papillary cell carcinoma (KIRP), liver hepatocellular carcinoma (LIHC), lung squamous cell carcinoma (LUSC), prostate adenocarcinoma (PRAD), rectum adenocarcinoma (READ), stomach adenocarcinoma (STAD), thyroid carcinoma (THCA), and uterine corpus endometrial carcinoma (UCEC).

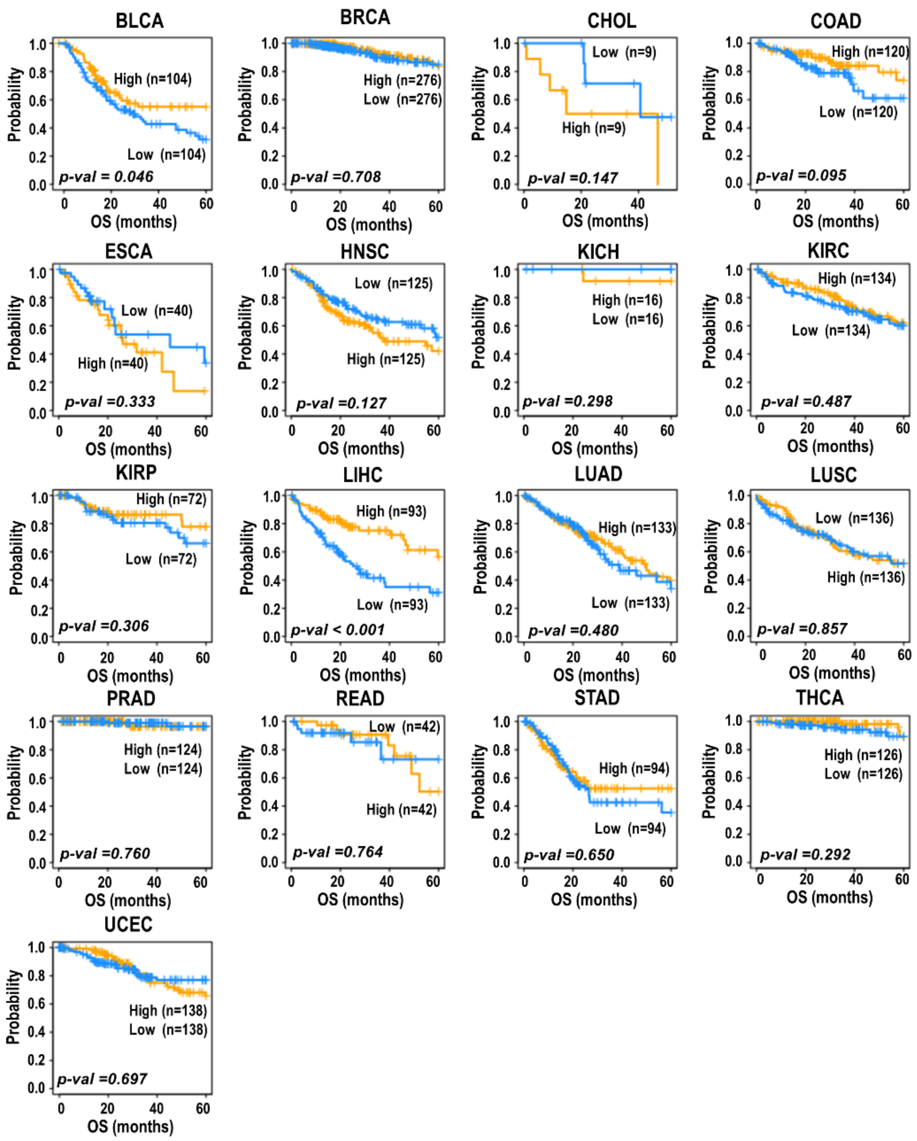

Suppl. Figure S4. Comparison of overall survival (OS) time between high and low MRPS31 expression groups of 17 different cancer types in TCGA.

The seventeen different type cancer cohorts from TCGA were stratified into high and low group based on the MRPS31 expression levels (above upper or below lower quartile of each cohort population). To analyze overall survival rates of the groups, KM survival curves was used. The survival probability at each time point, sample numbers of each group, and statistical p-values by the Cox-Mantel log-rank test are depicted.

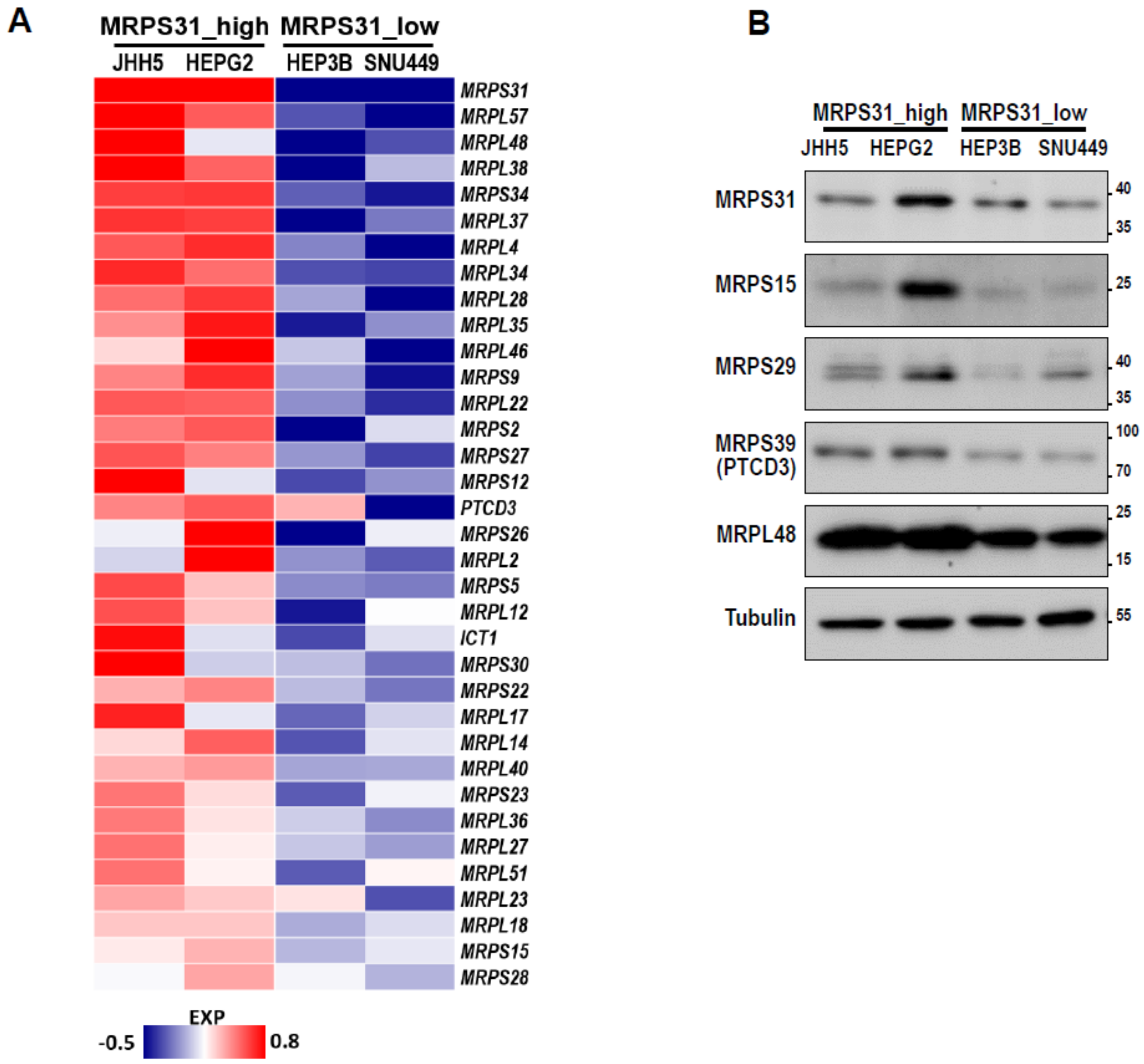

Suppl. Figure S5. Expression of MRPs in the four hepatoma cell lines.
(A) Heatmap of mRNA levels of MRPs. (B) Western blots.

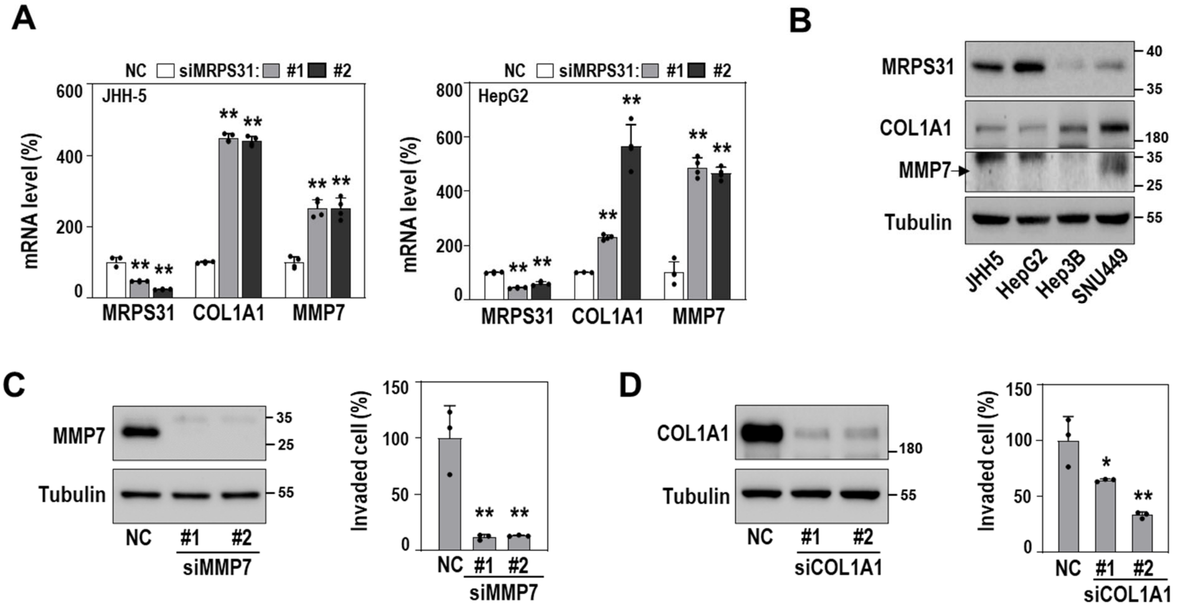

Suppl. Figure S6. Regulation of MMP7 and COL1A1 by MRPS31 knockdown and their involvement in cell invasion activity.

(A, B) Messenger RNA levels by qRT-PCR after MRPS31_high type cells, JHH5 (left) and HepG2 (right), were transfected with MRPS31 siRNA for 3days. (B) Western blots. (C, D) SNU449 (a MRPS31_low type cell) was transfected with siRNAs against MMP7 (C) and COL1A1 (D). Western blots (left) and cell invasion activity (right).
